# A Comparison Between Bisphosphonates and Teriparatide in the Treatment of Postmenopausal Osteoporosis: A Systematic Review

**DOI:** 10.7759/cureus.106378

**Published:** 2026-04-03

**Authors:** Russaal S Mann, Isha Chopra, Abdullah Kilic, Ayushi Saxena, Bilal Khan, Rupanshu Rupanshu, Paolo S Chavez Cavalie

**Affiliations:** 1 Internal Medicine, Vardhman Mahavir Medical College and Safdarjung Hospital, New Delhi, IND; 2 Anesthesia, Mahatma Gandhi Medical College and Hospital, Jaipur, IND; 3 Internal Medicine, Hackensack University Medical Center, Montclair, USA; 4 Medicine, California Institute of Behavioral Neurosciences and Psychology, Fairfield, USA; 5 Health Sciences, Queen's University, Kingston, CAN; 6 Internal Medicine, St. Martinus University Faculty of Medicine, Willemstad, CUW; 7 Surgery, Universidad Peruana de Ciencias Aplicadas, Lima, PER

**Keywords:** alendronate, antiresorptive, bisphosphonates, bone mineral density, fracture risk, osteoporosis, risedronate, romosozumab, systematic review, teriparatide

## Abstract

The majority of clinical research continues to focus on evaluating the efficacy of teriparatide and bisphosphonates in the treatment of osteoporosis. This systematic review gathered data from extensive research assessing the efficacy, safety, and clinical outcomes of various medications. The objective was to determine the effectiveness and clinical implications of teriparatide and bisphosphonates in the treatment of postmenopausal osteoporosis. We analyzed 15 comprehensive studies, encompassing randomized controlled trials (RCTs), systematic reviews, and meta-analyses. The primary studies included large trials and several medium-sized trials that examined changes in bone mineral density (BMD), fracture risk, and adverse outcomes. Teriparatide significantly lowered vertebral fracture risk compared with risedronate in patients with severe osteoporosis and increased lumbar spine BMD to a comparable extent as alendronate, though via a distinct anabolic mechanism. Combination therapy with zoledronic acid resulted in greater BMD gains than either agent alone. Most adverse events were mild and transient, including injection-site reactions, nausea, and dizziness, with no significant difference in serious adverse event rates compared with bisphosphonates. Network meta-analyses indicate that romosozumab may achieve greater early spine BMD gains than teriparatide.

Teriparatide is effective in lowering vertebral fracture risk and enhancing BMD in postmenopausal osteoporosis. Anabolic agents, including teriparatide, are recommended as first-line therapy for patients at very high risk of fractures (e.g., very low BMD with prevalent fractures or fractures occurring during glucocorticoid therapy), followed by a transition to antiresorptive therapy. Treatment selection should be guided by guideline-based risk stratification rather than applying a uniform stepwise approach.

## Introduction and background

Osteoporosis is a systemic skeletal disorder characterized by low bone mass and deterioration of bone microarchitecture, leading to increased bone fragility and fracture susceptibility [1]. The disease affects an estimated 200 million people worldwide, with a prevalence of approximately 20% in the general population and markedly higher rates among postmenopausal women. Postmenopausal women are disproportionately affected due to accelerated bone loss after estrogen decline [2]. Notable racial and ethnic disparities exist: White women have significantly higher fracture rates than Black women, though osteoporosis affects all populations and remains underdiagnosed across racial and ethnic groups. Osteoporotic fractures, particularly hip and vertebral fractures, are associated with substantial morbidity and excess mortality. Post-fracture mortality rates, especially following hip fracture, can match or exceed those observed after major cardiovascular events in some populations, underscoring the critical importance of effective treatment [3].

The pharmacological management of osteoporosis comprises two principal classes: antiresorptive agents, which suppress osteoclast-mediated bone resorption, and anabolic agents, which promote osteoblast-mediated bone formation. Antiresorptive agents, including the oral bisphosphonates alendronate, risedronate, and ibandronate; the intravenous bisphosphonate zoledronic acid; and the RANKL inhibitor denosumab, have long been the mainstay of osteoporosis treatment [1,4]. Teriparatide, a recombinant parathyroid hormone fragment approved over two decades ago, is an established anabolic agent primarily indicated for patients with severe osteoporosis or at very high fracture risk [5]. More recently, romosozumab, a sclerostin inhibitor with dual anabolic and antiresorptive properties, has further expanded the therapeutic armamentarium [6].

Current clinical guidelines provide risk-based recommendations for treatment selection: antiresorptive agents are indicated for patients at high fracture risk, while anabolic agents (including teriparatide) are recommended as first-line therapy for patients at very high risk, such as those with very low bone mineral density (BMD), prevalent severe or multiple vertebral fractures, a recent fracture, or fractures occurring during glucocorticoid therapy, followed by a sequential transition to antiresorptive therapy [5,7,8]. While these guidelines are well established, ongoing research continues to refine the understanding of comparative efficacy across specific patient populations, optimal treatment sequencing, and the influence of prior therapy on subsequent treatment responses [3,8].

This review aimed to synthesize current evidence comparing teriparatide with bisphosphonates, focusing on their effects on BMD and fracture risk reduction [9,10]. Drawing upon recent randomized controlled trials (RCTs) and systematic reviews, we sought to provide clinicians with an evidence-based overview to support guideline-directed treatment decisions, with particular attention to safety profiles, treatment adherence, and identifying patient populations most likely to benefit from each therapeutic approach [7,11].

## Review

Methodology

Search Strategy and Study Selection Process

We conducted a comprehensive literature search in compliance with the Preferred Reporting Items for Systematic Reviews and Meta-Analyses (PRISMA) 2020 criteria. We employed a Boolean method to search for pertinent articles regarding the efficacy of teriparatide in comparison to bisphosphonates in postmenopausal osteoporosis. We used a mix of Medical Subject Headings (MeSH) phrases and keywords like "Teriparatide," "Bisphosphonates," and "postmenopausal osteoporosis." The databases that were used were PubMed/MEDLINE, ScienceDirect, and the New England Journal of Medicine. They examined both free and paid full-text articles published from 2020 to 2025. The trial population comprised patients diagnosed with postmenopausal osteoporosis from various age groups, genders, and nationalities, all undergoing treatment with teriparatide and bisphosphonates. Table 1 presents the details of the search strategy and its results.

**Table 1:** Search stratgey details PubMed Central is a free full-text archive of biomedical and life sciences journal literature at the US National Institutes of Health's National Library of Medicine. ScienceDirect is the world's leading source for scientific, technical, and medical research. The Cochrane Library is a pre-filtered source that offers access to either synthesized publication types or critically appraised and carefully selected references MeSH: Medical Subject Headings; NEJM: The New England Journal of Medicine

Search strategy (keywords)	Database used	Number of papers identified
Teriparatide AND Bisphosphonates AND postmenopausal osteoporosis	PubMed	82
((( "Osteoporosis, Postmenopausal/prevention and control" [MeSH] OR "Osteoporosis, Postmenopausal/therapy"[MeSH] )) AND "Teriparatide/therapeutic use" [MeSH] AND ("Diphosphonates/pharmacology"[MeSH] OR "Diphosphonates/therapeutic use"[MeSH] )	PubMed (MeSH)	41
((((Teriparatide[Title/Abstract]) AND (Bisphosphonates [Title/Abstract])) AND (postmenopausal osteoporosis [Title/Abstract])) AND (English [Language])) AND (("2020/01/01"[Date - Publication] : "3000"[Date - Publication]))	PubMed	20
Teriparatide AND Bisphosphonates AND postmenopausal osteoporosis	NEJM	1
Teriparatide AND Bisphosphonates AND postmenopausal osteoporosis	ScienceDirect	863
Teriparatide AND Bisphosphonates AND postmenopausal osteoporosis	Cochrane	11

**Figure 1:**
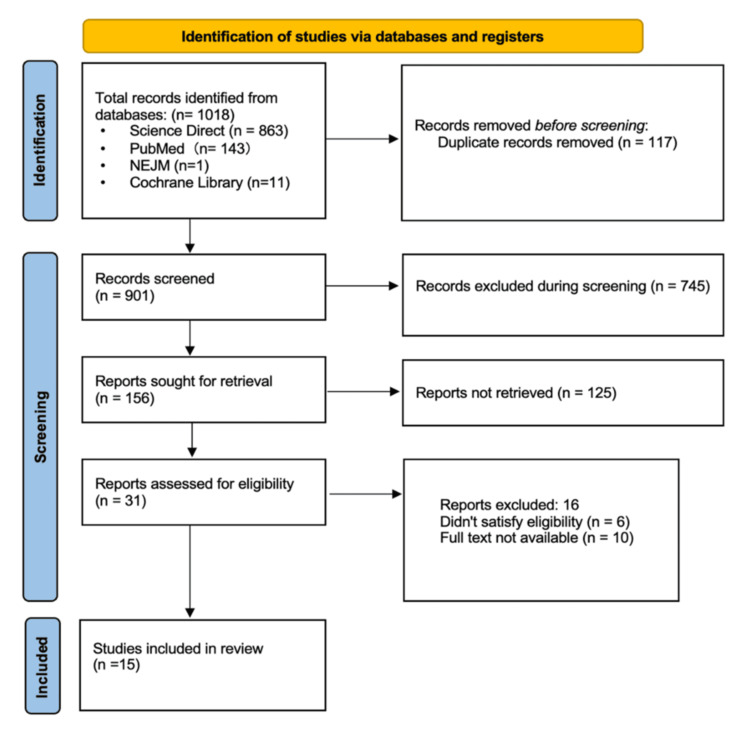
PRISMA flowchart depicting the process of article selection PRISMA: Preferred Reporting Items for Systematic Reviews and Meta-Analyses

Utilizing standard keywords, ScienceDirect, the New England Journal of Medicine, and PubMed identified 54 pertinent articles. The vetting process involved identifying and eliminating duplicate records, as well as excluding all gray literature and studies involving animals. A total of 1,018 studies were identified across multiple databases: ScienceDirect (863), PubMed (143), and the New England Journal of Medicine (1). Following the removal of 117 duplicate entries and 745 records for other reasons, a total of 156 records remained. After reviewing the titles and abstracts, we identified 31 studies that contributed to the research and 16 that did not. Following a comprehensive full-text review, only 15 articles fulfilled the requisite criteria for this research subject. Figure 1 presents the PRISMA flowchart illustrating the literature selection process and the methodology employed in searching for information.

Inclusion and Exclusion Criteria

Multiple criteria were evaluated for the study. The review focused on RCTs conducted from 2020 to 2025. The primary focus was on patients aged 45 years and older. We included studies involving female patients who received either teriparatide, bisphosphonates, or a combination of both therapies. All investigations were exclusively limited to randomized controlled trials conducted with human participants. We excluded studies classified as grey literature and unpublished research, as well as animal studies. Attention was also given to identifying and removing duplicate records. Table 2 presents the criteria for inclusion and exclusion in the study.

**Table 2:** Inclusion and exclusion criteria

Inclusion criteria	Exclusion criteria
Articles published in the past five years	Gray literature
Middle-aged patients: 45 years and above	Unpublished literature
Female patients	Animal studies
Full-text articles	Studies that focus on other drugs
Studies with patients using at least one of the two drugs	
Papers published in English	

The systematic data extraction involved 15 full-text articles, including five RCTs, eight systematic reviews/meta-analyses, and two comparative studies. The principal data extracted from the studies included study parameters (design, population size, treatment regimens), primary outcomes (BMD changes, fracture rates), and safety variables (adverse events, discontinuation rates). We gained a deeper understanding of how to search, what to include, and how to assess the quality of meta-analyses and systematic reviews. The extraction process focused on three main types of results: safety and adverse events, outcomes related to bone mineral density, and reduction in fracture risk. We organized the data into standard tables so that it would be easier to review and compare studies. When available, we paid close attention to statistical indicators such as odds ratios, relative risks (RRs), and confidence intervals (CIs).

Results

Quality Assessment

A comprehensive quality assessment was conducted using standardized instruments appropriate for each study type. The AMSTAR 2 assessment of the systematic reviews and meta-analyses indicated that seven studies employed high-quality methodologies. The only review that received an average grade failed to register protocols, perform duplicate study selection, or assess publication bias. The high-quality reviews demonstrated the utilization of robust methodologies, including rigorous risk-of-bias assessments, exhaustive search strategies, and duplicate data extraction. The Cochrane Risk of Bias 2 (RoB 2) tool identified two RCTs exhibiting a low risk of bias across all domains. However, three trials raised some concerns because they deviated from planned interventions or had incomplete outcome data. In general, none of the RCTs were considered to have a high risk of bias. The observational studies were rated using the Newcastle-Ottawa Scale (NOS). The clinical comparative study received a high-quality rating (8 out of 9 stars), while the retrospective study received a moderate-quality rating (7 out of 9 stars). Both observational studies demonstrated strong selection and comparability attributes; however, they differed in the completeness of follow-up. The quality assessment indicates a robust body of evidence overall. The majority of the studies met stringent methodological criteria for their design categories, thereby reinforcing the reliability of the findings for clinical decision-making. Table 3 presents the AMSTAR 2 quality assessment of systematic reviews/meta-analyses.

**Table 3:** AMSTAR 2 quality assessment of systematic reviews/meta-analyses Quality rating categories: high: no or one non-critical weakness; moderate: more than one non-critical weakness; low: one critical flaw with or without non-critical weaknesses; critically low: more than one critical flaw with or without non-critical weaknesses PICO: Population (or Patient/Problem), Intervention, Comparison (or Control), and Outcome; AMSTAR: A MeaSurement Tool to Assess systematic Reviews; RoB: Risk of Bias; Y: yes; PY: partial yes; N: no; NA: not applicable

AMSTAR 2 criteria	Li et al., 2024 [4]	Nunkoo et al., 2024 [7]	Willems et al., 2022 [3]	Wang et al., 2023 [1]	Yang et al., 2024 [2]	Jeon et al., 2024 [8]	Arthur Vithran et al., 2024 [10]	Ebina et al., 2024 [6]
1. PICO components	Y	Y	Y	Y	Y	Y	Y	Y
2. Protocol registration	Y	Y	Y	Y	Y	Y	Y	N
3. Study design selection explained	Y	Y	Y	Y	Y	Y	Y	PY
4. Comprehensive search	Y	Y	Y	Y	Y	Y	Y	PY
5. Duplicate study selection	Y	Y	Y	Y	Y	Y	Y	N
6. Duplicate data extraction	Y	Y	Y	Y	Y	Y	Y	N
7. List of excluded studies	PY	PY	Y	Y	PY	PY	PY	N
8. Detailed study description	Y	Y	Y	Y	Y	Y	Y	Y
9. RoB assessment	Y	Y	Y	Y	Y	Y	Y	PY
10. Funding sources reported	Y	Y	Y	Y	Y	Y	Y	Y
11. Appropriate statistical methods	Y	Y	Y	Y	Y	Y	Y	NA
12. RoB impact on meta-analysis	Y	Y	Y	Y	Y	Y	Y	NA
13. RoB in individual studies	Y	Y	Y	Y	Y	Y	Y	PY
14. Heterogeneity explanation	Y	Y	Y	Y	Y	Y	Y	PY
15. Publication bias assessment	Y	Y	Y	Y	Y	Y	Y	N
16. Conflicts of interest	Y	Y	Y	Y	Y	Y	Y	Y
Overall quality rating	High	High	High	High	High	High	High	Moderate

Table 4 shows the Newcastle-Ottawa Scale assessment details of observational studies.

**Table 4:** Newcastle-Ottawa Scale assessment

Criteria	Maximum stars	Zidrou and Beletsiotis, 2024 [12]	Gou et al., 2021 [13]
Selection	4	3	3
1. Representativeness of exposed cohort	1	★	★
2. Selection of non-exposed cohort	1	★	★
3. Ascertainment of exposure	1	★	★
4. Demonstration that the outcome was not present at the start	1	☆	☆
Comparability	2	2	2
1. Study controls for the most important factor	1	★	★
2. Study controls for additional factors	1	★	★
Outcome	3	3	2
1. Assessment of outcome	1	★	★
2. Was the follow-up long enough for outcomes	1	★	★
3. Adequacy of follow-up of cohorts	1	★	☆
Total score	9	8	7
Quality rating		High	Good

Table 5 presents the Cochrane Risk of Bias 2 (RoB 2) assessment for RCTs.

**Table 5:** Cochrane Risk of Bias 2 (RoB 2) assessment for RCTs RCTs: randomized controlled trials

Domain	Geusens et al., 2018 [5]	Body et al., 2020 [9]	Li et al., 2022 [11]	Takeuchi et al., 2024 [14]	Hagino et al., 2021 [15]
1. Randomization process	Low	Low	Low	Low	Low
2. Deviations from intended interventions	Low	Low	Some concerns	Some concerns	Some concerns
3. Missing outcome data	Low	Low	Low	Low	Some concerns
4. Measurement of outcome	Low	Low	Low	Low	Low
5. Selection of reported results	Low	Low	Some concerns	Low	Low
Overall risk of bias	Low	Low	Some concerns	Some concerns	Some concerns

Study Design Overview

Table 6 provides* *an overview of the included studies

**Table 6:** Overview of the included studies BMD: bone mineral density; CI: confidence interval; RR: relative risk

Study	Study type	Sample size	Intervention	Follow-up period	Outcomes measured	BMD change, spine	BMD change, hip	Vertebral fractures	Relative risk or equivalent	Types of adverse events	Frequency	Severity	Discontinuation rate
Wang et al., 2023 [1]	Systematic review/meta-analyses	39,095	Denosumab, teriparatide, zoledronic acid, ibandronic acid		Fracture, BMD, safety	Mean difference: 4.64 (spine), 95% CI: 1.60-7.72		Compared with placebo, denosumab (RR: 0.325; 95% CI: 0.149–0.706, zoledronic acid (RR: 0.353; 95% CI: 0.218–0.593), and teriparatide (RR: 0.360; 95% CI: 0.238–0.505 significantly reduced the incidence of vertebral fractures	Significant	Arthralgia, nasopharyngitis, back pain	Among patients receiving denosumab, 95.1% experienced adverse events, most commonly arthralgia, nasopharyngitis, back pain, and so on. In Boonen's study, 30% of patients receiving teriparatide, 83% experienced multiple adverse events, including asthenia, arrhythmia, and hypertension	There was no significant difference in serious adverse events among the four anti-osteoporosis drugs	
Yang et al., 2024 [2]	Systematic review/meta-analyses		Teriparatide, denosumab vs. oral bisphosphonates	12 months	BMD changes, safety	Increase in lumbar spine (RR: 5.16, 95% CI: 5.09–5.24)	Increase in femoral neck (denosumab)						
Willems et al., 2022 [3]	Systematic review/meta-analyses		Multiple osteoporosis drugs	12–36 months	Fracture, BMD outcomes								
Li et al., 2024 [4]	Systematic review/meta-analyses	6,680	Teriparatide/denosumab vs. bisphosphonates	>12 months	Fracture risk, BMD changes	Increase in BMD (all sites)	Increase in BMD (all sites)	The results of the meta-analysis showed that teriparatide was superior to bisphosphonates in decreasing the risk of fracture (RR: 0.61, 95% CI: 0.51–0.74)	Significant				
Geusens et al., 2018 [5]	Randomized controlled trial	1,360	Teriparatide 20 micrograms daily subcutaneous vs. risedronate 35 mg weekly oral, 24 months	24 months	New vertebral fractures, clinical fractures			The treatment effect observed across the entire study population showed an incident rate of new vertebral fractures of 5.4% in the teriparatide group, compared to 12.0% in the risedronate group (RR: 0.44; 95% CI 0.29–0.68; p = 0.000094).	The treatment effect observed across the entire study population showed an incident rate of new vertebral fractures of 5.4% in the teriparatide group, compared to 12.0% in the risedronate group (RR: 0.44; 95% CI: 0.29–0.68; p = 0.000094)				
Ebina et al., 2024 [6]	Systematic review/meta-analyses		Teriparatide, abaloparatide, romosozumab	Up to 24 months	BMD, fracture reduction	Increases in BMD from baseline, with an 8.6% enhancement in the lumbar spine compared to placebo	3.6% increase in total hip (TH) compared to placebo	In the phase III Pivotal Fracture Trial (PFT), involving a high fracture risk cohort of 1,637 postmenopausal women with previous vertebral fractures, daily administration of 20-μg teriparatide yielded a remarkable 65% reduction in the relative risk of vertebral fractures and a 35% reduction in non-vertebral fractures compared to placebo		Hypercalcemia, dizziness, nausea	Adverse events, including nausea, vomiting, and pyrexia, occurred at a substantially lower rate in the 28.2-μg twice-weekly group compared to the 56.5-μg once-weekly group (39.7% versus 56.2%; p < 0.01)	Although the risks of atypical femoral fractures and osteonecrosis of the jaw were similar between the two groups, romosozumab was linked to a higher incidence of severe cardiovascular events relative to alendronate	
Nunkoo et al., 2024 [7]	Systematic review/meta-analyses		Teriparatide vs. alendronate	72 weeks	BMD, vertebral fracture, quality of life	Increase in lumbar spine and hip (teriparatide)	Lower increase in hip (alendronate)	The annual incidence rate of morphometric vertebral fracture in the sequential therapy (teriparatide) group (0.1020 and 0.1334) was significantly lower than that of monotherapy (alendronate) (0.1492 and 0.1734)	Significant	Back pain, headache, dizziness, nausea			
Jeon et al., 2024 [8]	Systematic review/meta-analyses		Anabolic agents vs. bisphosphonates		New osteoporotic vertebral fracture, fracture healing			In patients with prevalent OVF, anabolic agents significantly reduced the incidence of new OVF (teriparatide and romosozumab vs. alendronate and risedronate (RR: 0.57; 95% CI: 0.45–0.71; p < 0.00001; high-certainty of evidence); teriparatide vs. risedronate (RR: 0.50; 95% CI: 0.37–0.68; p < 0.0001; high-certainty of evidence)	Significant				
Body et al., 2020 [9]	Randomized controlled trial	1,360	Teriparatide 20 micrograms daily subcutaneous vs. risedronate 35 mg weekly oral	24 months	FRAX^®^-defined major osteoporotic fractures			2.6% vs. 6.4% (major osteoporotic fracture)	In total, 16 patients (cumulative incidence: 2.6%) had one or more low-trauma FRAX ® - defined MOF in the teriparatide group compared with 40 patients (cumulative incidence: 6.4%) in the risedronate group (overall HR: 0.40; 95% CI: 0.23–0.68; p = 0.001)				
Arthur Vithran et al., 2024 [10]	Systematic review/meta-analyses		Teriparatide vs. other treatments		BMD, fracture, adverse events	Mean difference: 0.02 (spine), 95% CI: 0.01–0.03	Mean difference: 0.01 (femoral neck), 95% CI: 0.00–0.01	The synthesized results demonstrated a substantial decrease in fracture risk within the teriparatide group relative to the control group, with an RR of 0.57 and a 95% CI spanning from 0.45 to 0.72	Significant		The likelihood of adverse events increased RR: 1.65, 95% CI: 1.32–2.07		
Li et al., 2022 [11]	Randomized controlled trial	587	Teriparatide 20 micrograms daily subcutaneous vs. alendronate 70 mg weekly oral	48 weeks	Lumbar spine BMD change, fracture incidence	Non-inferior to alendronate (difference: 0.7%, 95% CI: −0.3 to 1.7%)	−1.0% (teriparatide), 2.2% (alendronate)	The incidence of new fractures showed no statistical difference between groups (P= = 0.128)	Not significant	Hypercalcemia, alkaline phosphatase increase, dizziness, arthralgia	76.5% (teriparatide), 63.9% (alendronate)	9.5% (serious, teriparatide), 4.6% (alendronate)	2.4% (teriparatide), 0.5% (alendronate)
Zidrou and Beletsiotis, 2020 [12]	Comparative trial		Zoledronic acid, teriparatide, or both	52 weeks	BMD, bone turnover, fractures	At week 52, lumbar spine BMD showed increases of 7.5%, 7.0%, and 4.4% in the combination, teriparatide, and zoledronic acid groups, respectively	Total hip BMD increases were 2.3%, 1.1%, and 2.2% across the respective subgroups	Clinical fractures, reported as adverse events, were documented in 7, 2, and 4 participants within the subgroups receiving zoledronic acid, combined treatment, and teriparatide, respectively		Nausea, flu-like, chills, fever	90.8% (combination/zoledronic acid), 86.1% (teriparatide)	There were no notable differences in the incidence of severe adverse events among the subgroups	Adverse effects leading to study discontinuation were observed in 6 patients (8.8%) within the combination treatment subgroup, in 4 patients (5.7%) within the teriparatide-only subgroup, and in 3 patients (4.3%) within the zoledronic acid-only subgroup
Gou et al., 2021 [13]	Retrospective-comparative	53	Teriparatide vs. alendronate	12 months	Vertebral collapse, BMD, bone turnover markers	Increased significantly from baseline (0.65 AE 0.02 g/cm^2^) to month 12 (0.72 AE 0.01 g/cm^2^) in the teriparatide group (p < 0.001), and increased significantly from baseline (0.65 AE 0.02 g/cm^2^) to month 12 (0.69 AE 0.02 g/cm^2^) in the alendronate group (p < 0.001)							
Takeuchi et al., 2024 [14]	Randomized controlled trial	966	Once-weekly teriparatide vs. alendronate	72–120 weeks	Treatment discontinuation	The mean lumbar spine BMD (T-score) was comparably increased up to 72 weeks in both treatment groups				Gastrointestinal disorders	Gastrointestinal disorders are well-known adverse events associated with bisphosphonates, including ALN, and were observed in 9.4% of the ALN group in the JOINT-05 trial		
Hagino et al., 2021 [15]	Randomized controlled trial		Once-weekly teriparatide vs. alendronate	72 weeks	Morphometric vertebral fractures	Similar lumbar spine BMD elevation		The occurrence of morphometric vertebral fractures was significantly reduced in the teriparatide group (56 per 419.9 person-years, annual incidence rate: 0.1334) compared to the alendronate group (96 per 553.6 person-years, annual incidence rate: 0.1734), with a rate ratio of 0.78 (95% CI: 0.61 to 0.99, p = 0.04)	RR: 0.78 (95% CI: 0.61–0.99)	Infections, gastrointestinal, musculoskeletal			During the course of the trial, 238 patients in the teriparatide group and 139 patients in the alendronate group withdrew from the study. Among these, 142 patients in the teriparatide group and 70 patients in the alendronate group elected to discontinue the study treatment. Furthermore, 42 patients in the teriparatide group and 18 patients in the alendronate group discontinued the study treatment due to safety concerns

Discussion

Summary of Main Findings

This systematic review of 15 full-text studies provides evidence on the comparative effectiveness of teriparatide versus bisphosphonates in the treatment of postmenopausal osteoporosis. The principal findings demonstrate that teriparatide is more effective than risedronate in reducing the risk of vertebral fractures (5.4% vs. 12.0%, RR: 0.44, 95% CI: 0.29-0.68) [5] and major osteoporotic fractures (2.6% vs. 6.4%, hazard ratio: 0.40, 95% CI: 0.23-0.68) [9]. Regarding bone density outcomes, teriparatide and alendronate produced similar lumbar spine BMD gains, with a between-group difference of 0.7% (95% CI: −0.3 to 1.7%) [11]. Meta-analytic evidence supports these findings, with systematic reviews consistently demonstrating relative risk reductions for vertebral fractures ranging from 0.47 to 0.61 [4,7,10].

Interpretation in the Context of Existing Literature

The superior reduction of vertebral fractures associated with teriparatide is consistent with its anabolic mechanism of action, which stimulates new bone formation rather than solely inhibiting bone resorption [1]. This mechanistic advantage is particularly evident at the spine, where trabecular bone predominates and is more responsive to anabolic stimulation [6]. These findings align with broader evidence that anabolic agents reduce vertebral fracture risk by 65-86%, compared with 50-70% for antiresorptive agents [6]. While our primary review question concerns teriparatide versus bisphosphonates, several included network meta-analyses [1,3,8] also evaluated other agents. Notably, romosozumab has demonstrated spine BMD gains of 13.3% compared with 8.6% for teriparatide [3,6], suggesting that the therapeutic landscape continues to evolve.

Clinical Implications and Treatment Recommendations

Current international guidelines provide clear risk-stratified recommendations for osteoporosis pharmacotherapy [16,17]. For patients at high fracture risk, oral bisphosphonates remain an appropriate and cost-effective first-line option [2]. For patients at very high fracture risk - defined by the The American Association of Clinical Endocrinology and the American College of Endocrinology (AACE/ACE) guidelines as those with a T-score ≤ −2.5 with fracture, T-score ≤ −3.0 (with or without fracture), very high FRAX probability, recent fracture (within 12 months), fractures occurring on approved osteoporosis therapy, or multiple vertebral fractures - anabolic agents including teriparatide are recommended as initial therapy, followed by sequential transition to antiresorptive treatment [16,17]. The European guidance similarly supports the use of teriparatide as a first-line agent in women with severe osteoporosis at very high fracture risk [16]. The annual incidence of vertebral fractures (0.1334 for teriparatide vs. 0.1734 for alendronate) [15] and network meta-analyses consistently ranking teriparatide among the most effective interventions for vertebral fracture prevention [1,8] further support this recommendation.

Safety Considerations and Tolerability

The safety profile of teriparatide requires careful interpretation. While the overall incidence of any adverse events was numerically higher with teriparatide than alendronate (76.5% vs. 63.9%) [11], the majority of these events were mild and transient - most commonly injection-site reactions, transient dizziness, nausea, and headache - and are not considered clinically significant [7,10,16]. Serious adverse events were rare and did not differ significantly between teriparatide and bisphosphonates across most included studies [7,10,12]. Treatment-limiting adverse events leading to discontinuation occurred at low rates (2.4% for teriparatide vs. 0.5% for alendronate in one trial [11]), and clinical experience indicates that teriparatide is generally well tolerated in practice [16].

An important safety consideration is that teriparatide therapy is limited to a maximum of 24 months over a patient's lifetime, due to a theoretical risk of osteosarcoma identified in preclinical rodent studies. This lifetime treatment limitation is reflected in regulatory labeling and is endorsed by current clinical guidelines [16,17]. Routine monitoring of serum calcium levels during treatment is recommended [3,11].

Economic Considerations and Cost-Effectiveness

Although comprehensive cost-effectiveness evaluations were limited in the included studies, the substantially higher cost of teriparatide relative to bisphosphonates remains an important factor in treatment decisions [1]. The economic burden of osteoporotic fractures is considerable, and the additional cost of teriparatide may be justified in patients at very high fracture risk given its superior fracture prevention efficacy [16,17]. Further pharmacoeconomic studies incorporating quality-adjusted life years and long-term fracture prevention benefits are needed to guide resource allocation.

Subgroup Analyses and Patient Selection

The evidence suggests differential treatment responses across patient subgroups. Prior antiresorptive treatment may attenuate the BMD response to teriparatide, although the medication remains effective in bisphosphonate-pretreated patients [7]. Teriparatide's efficacy is particularly pronounced in patients with severe osteoporosis and multiple risk factors [5,9], supporting its preferential use in this population. Age-related considerations are relevant, as older patients may derive the greatest absolute benefit from fracture prevention while warranting closer monitoring [14].

Combination and Sequential Therapy Approaches

Evidence suggests potential benefits of combination therapy strategies. Zidrou and Beletsiotis reported that combination therapy with teriparatide and zoledronic acid produced a 7.5% increase in spine BMD compared with 7.0% with teriparatide monotherapy [12]. Sequential therapy - anabolic treatment followed by antiresorptive consolidation - may optimize long-term outcomes by building bone during the anabolic phase and preserving gains thereafter [8,16,17]. Further research is needed to determine the optimal timing, duration, and sequencing of these approaches.

Strengths and limitations

This review has several methodological strengths, including the incorporation of both individual RCTs and meta-analyses, comprehensive quality assessment using validated tools (AMSTAR 2, RoB 2, NOS), and substantial combined sample sizes [1]. However, it has certain limitations as well, including heterogeneity in study designs and outcome measures, potential selection bias from restricting to full-text articles, limited long-term data beyond 24 months, and a focus on postmenopausal women that limits generalizability to other populations.

Future research directions

Several critical knowledge gaps warrant further investigation. Long-term comparative efficacy trials exceeding 24 months are essential to determine the durability of therapeutic effects and long-term safety profiles [8]. Head-to-head comparisons of teriparatide with newer anabolic agents such as romosozumab and abaloparatide are needed to inform treatment selection in an evolving therapeutic landscape [6]. Research should also focus on identifying optimal treatment sequences and the timing of transitions from anabolic to antiresorptive therapy, including the development of evidence-based algorithms for sequential therapy [10,16,17].

Real-world effectiveness studies are needed to validate clinical trial findings in routine practice, including data on adherence patterns, discontinuation rates, and outcomes in diverse patient populations [2]. Pharmacogenomic research may identify genetic markers that predict individual treatment response and adverse events, enabling more personalized therapy selection. Cost-effectiveness evaluations incorporating quality-adjusted life years and long-term fracture prevention benefits are necessary to guide healthcare policy and reimbursement decisions.

The development of biomarkers predictive of treatment response represents another important research priority. Additionally, determining the optimal duration of anabolic therapy and strategies to maintain bone formation gains after treatment completion remain key areas for improving long-term patient outcomes [4].

## Conclusions

This systematic review demonstrates that teriparatide is effective in reducing vertebral fracture risk and improving BMD in postmenopausal osteoporosis. Consistent with AACE/ACE and European guidelines, teriparatide is recommended as an initial therapy for patients at very high fracture risk, including those with severe osteoporosis with or without existing fractures, followed by a sequential transition to antiresorptive therapy. The safety profile is generally favorable, with most adverse events being mild and transient, though therapy is limited to 24 months based on preclinical safety considerations. Treatment decisions should be guided by established risk stratification, individual patient characteristics, and current clinical guidelines.
